# The effect of a novel low temperature-short time (LTST) process to extend the shelf-life of fluid milk

**DOI:** 10.1186/s40064-016-2250-1

**Published:** 2016-05-31

**Authors:** Phillip R. Myer, Kyle R. Parker, Andrew T. Kanach, Tengliang Zhu, Mark T. Morgan, Bruce M. Applegate

**Affiliations:** Department of Animal Science, University of Tennessee, Knoxville, TN 37996 USA; Department of Biological Sciences, Purdue University, West Lafayette, IN 47907-2054 USA; Department of Food Science, Purdue University, West Lafayette, IN 47907-2009 USA; Food Science and Technology Department, University of Tennessee, Knoxville, TN 37996 USA

**Keywords:** Low temperature, LTST, MST, Pasteurization, BARDOT

## Abstract

Pasteurization has long been the standard method to extend the shelf-life of dairy products, as well as a means to reduce microbial load and the risk of food-borne pathogens. However, the process has limitations, which include cost effectiveness, high energy input, and reduction of product quality/organoleptic characteristics. In an effort to reduce these limitations and extend shelf-life, this study examined a novel low temperature, short time (LTST) method in which dispersed milk in the form of droplets was treated with low heat/pressure variation over a short treatment time, in conjunction with pasteurization. *Lactobacillus fermentum* and *Pseudomonas fluorescens* Migula were exposed to conventional pasteurization treatments with and without LTST. Using these organisms, the LTST addition was able to reduce microbial load below detection limits; 1.0 × 10^1^ cfu/mL, from approximately 1.2 × 10^8^ and 1.0 × 10^7^ cfu/mL for *L. fermentum* and *P. fluorescens* Migula, respectively. In addition, the shelf-life of the treated, raw, and uninoculated product was prolonged from 14 to 35 days, compared with standard pasteurization, to as long as 63 days with the LTST amendment. Sensory analysis of samples also demonstrated equal or greater preference for LTST + pasteurization treated milk when compared to pasteurization alone (α = 0.05). Conventional pasteurization was effective at reducing the above mentioned microorganisms by as much as 5.0 log10 cfu/mL. However, LTST was able to achieve 7.0–8.0 log10 cfu/mL reduction of the same microorganisms. In addition, BActerial Rapid Detection using Optical scattering Technology detected and identified microorganisms isolated both pre- and post-treatment, of which the only organisms surviving LTST were *Bacillus* spp. Increased lethality, improved shelf-life, and equal or better organoleptic characteristics without increased energy consumption demonstrate the effectiveness of the incorporation of LTST. The improved shelf-life may potentially have major impacts in the dairy industry in terms of shipping and overall sustainability.

**Electronic supplementary material:**

The online version of this article (doi:10.1186/s40064-016-2250-1) contains supplementary material, which is available to authorized users.

## Background

The manufacturing and distribution dynamics of the fluid milk industry are constantly impacted by the primary concern of prolonging shelf-life and improving safety. The process of heating milk for a predetermined time at a predetermined temperature (i.e. pasteurization) is aimed at reducing microbial load and addressing the previous issues (Nada et al. 2012). Unfortunately, many characteristics of the pasteurization process are neither effectively robust nor cost effective. Currently, the shelf-life of unopened, pasteurized milk is 8–14 days depending on the intensity of the treatment (Niamsuwan et al. 2011), and the process itself consumes a significant amount of energy. Together, with rising fuel costs, the industrial sector must find solutions to minimize energy and fuel consumption in order to cut expenditures and reduce their carbon footprint.

Thermal processing, such as pasteurization, has been the standard method to extend the shelf-life of dairy products. These high temperatures (70–120 °C) can damage and/or cause structural modifications to proteins within the product, leading to noticeable changes in sensorial characteristics (Siciliano et al. 2000). Lowering the thermal threshold, while still achieving adequate microorganism reduction, would be an advantageous scenario; reducing energy use/costs and increasing the overall quality of the product. Such treatments exist, such as low temperature long-time (LTLT) pasteurization, or Holder pasteurization. However, these treatments have been shown to degrade and damage many biochemical components, such as vitamins: C, folacin, and B_6_ (Van Zoeren-Grobben et al. 1987; Moltó-Puigmartí et al. 2011). High-pressure processing (HPP) is utilized as an alternative to thermal treatment, but proteins, enzymes, polysaccharides and nucleic acids have also been shown to be adversely affected (Balci and Wilbey 1999).

A viable process would reduce costs and energy input, improve the quality of the product, and maintain or improve microorganism reduction potential. Such benefits would have a tremendous impact on the industry, including: increased cost effectiveness, higher quality product, and increased distribution distances. Increased shelf-life would also allow for a decrease in distribution locations; distribution from a few locations, rather than hundreds.

This study examined a novel, low temperature, short time (LTST) amendment for pasteurization, in which low heat and variable pressure were utilized to aid in the pasteurization of a fluid milk product. This method, utilizing an Millisecond Technologies (MST) chamber unit and previously described (Arofikin 2010), was characterized by examining microbial reduction, shelf-life, and sensory evaluation in order to assess the efficacy of the treatment process. In addition, detection and identification of microorganisms isolated both pre- and post-treatment were conducted via BActerial Rapid Detection using Optical scattering Technology (BARDOT; Banada et al. 2009; Singh et al. 2014) and 16S rRNA gene sequencing, in order to determine the identity and characteristics of survivor organisms at differing steps in the process, allowing insight into the effect on potential spoilage organisms. We hypothesize that the LTST amendment will increase microbial reduction and shelf-life, while maintaining the quality and organoleptic characteristics of the product.

## Results and discussion

### LTST bacterial reduction

**Fig. 1:**
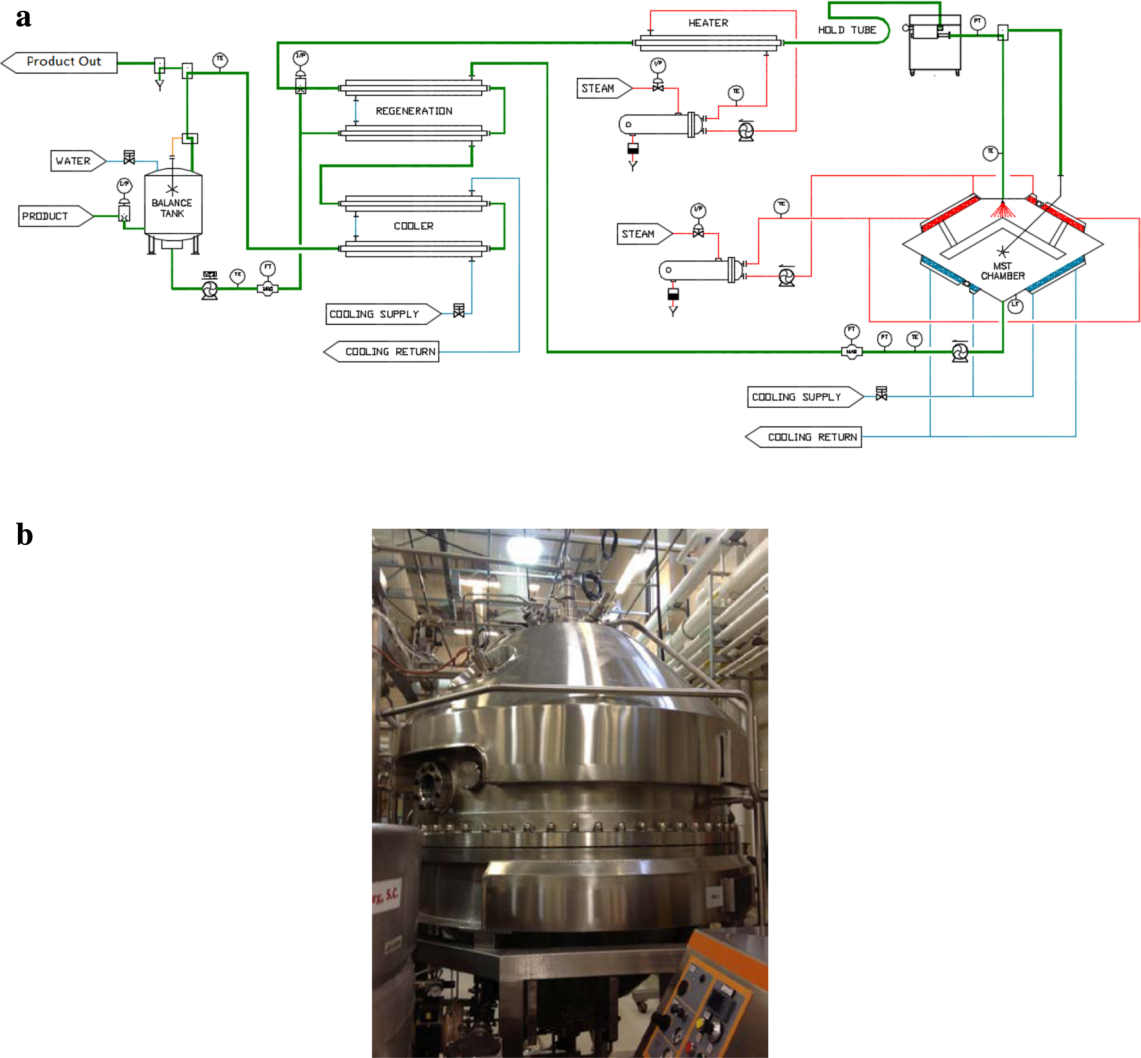
MST unit. **a** Schematic of the MST Process (LTST) or (MST). **b** Image of the MST unit (Millisecond Technologies, New York, NY)

The LTST method depends on a mechanism in which low heat and low pressure are utilized to pasteurize a fluid milk product (Arofikin 2010). In this process, milk is dispersed in the form of droplets into a process chamber and heated for 0.02 s at or below pasteurization temperatures (≤72.7 °C). The MST chamber schematic and image are depicted in Fig. 1.

In order to assess the efficacy of the LTST process, the logarithmic reduction of bacterial species was examined utilizing model Gram-positive and -negative organisms; *Lactobacillus fermentum* and *Pseudomonas fluorescens* Migula, respectively. Tolerance to heat varies between organisms and thus, the extent of the pasteurization treatment is determined by the thermal characteristics of its model organisms, and time–temperature relationships must be examined. Additional file 1: Fig. S1 shows the D- and Z-values for *P. fluorescens* Migula, while Additional file 1: Fig. S2 shows similar information for *L. fermentum.* The values demonstrate the greater thermal resistance of *L. fermentum* compared to that of *P. fluorescens* Migula. With a Z-value 9.00, compared to 7.09, *L. fermentum* is able to withstand an increase in thermal treatment greater than *P. fluorescens* Migula. This is further demonstrated by the associated D-values: D_60_ = 4.92 and 44.29 s, for *P. fluorescens* Migula and *L. fermentum* respectively. The results also demonstrate the capability to assess the LTST method using these organisms as a model, due to the variance in their thermal characteristics.

Utilizing *P. fluorescens* Migula and *L. fermentum*, the LTST amendment was applied to assess the microbial reduction potential of the system. The sampling model is depicted in Additional file 1: Fig. S3. The first two trials assessed the system, using process temperatures at or near that of pasteurization temperatures (Table 1). The results show that in most runs, significant populations (P < 0.05) of the inoculated microorganisms survived pasteurization conditions (Table 1), but did not survive the MST unit treatment which followed. For Trial 1, Run 1, the natural microbiota in the raw milk was approximately 10^4^ cfu/ml and the pasteurization conditions were enough to fully eliminate these organisms. However, for Runs 2 and 3 in the first trial, *L. fermentum* survived standard pasteurization. This was especially evident when compared to that of the natural microbiota, as only 1–2 log10 (90–99 %) cfu/mL of the microorganisms were eliminated due to similar pasteurization conditions. The organisms that survived pasteurization were reduced by the MST unit which followed, when operated under the conditions listed in Table 1B.

**Table 1:** LTST microbial reduction

(A)	Run	Indicator organism	Micro counts (cfu/mL)		
Trial			Before processing (cfu/mL)	After pasteurizer (cfu/mL)^1^	After MST (cfu/mL)
1	1	None	5.7 × 10^4 a^	NS^b^	NS^b^
	2	*L. fermentum*	1.2 × 10^8 a^	5.0 × 10^6 b^	NS
	3	*L. fermentum*	1.2 × 10^8 a^	1.2 × 10^7 b^	NS
2	1	*P. fluorescens* Migula	6.4 × 10^6 a^	NS^b^	NS^b^
	2	*P. fluorescens* Migula	9.0 × 10^6 a^	3.4 × 10^5 b^	NS^c^
	3	*P. fluorescens* Migula	1.2 × 10^7 a^	1.1 × 10^6 b^	NS^c^

(B)	Trial 1^2^			Trial 2^2^		
Run	1	2	3	1	2	3
MST Temp In^3^ (°C)	61.0	61.5	61.6	60.1	55.8	51.3
MST Chamber (°C)	73.7	73.7	75.7	74.1	71.2	68.3
MST Temp Out (°C)	74.6	74.3	76.2	69.7	66.7	62.9

A) The reduction of organisms inculcated into raw, prior-treated, milk before processing, after pasteurization, and after pasteurization + MST treatment. B) Process temperatures

^a,b,c^ Means among the groups were compared using ANOVA and the Tukey’s Test. Within a row, means with a different superscript were different (P < 0.05)

^1^NS = No significant microorganisms recovered; populations were below the detection limit (10^1^ cfu/mL)

^2^Process temperature (°C) means are of three readings (beginning, middle, and end) of each 30 min total run

^3^MST Temp In is approximately the same as pasteurizer temperature out

For Trial 2, the conditions were less severe, as LTST temperatures were reduced, including the product entering the MST unit and the MST chamber (Table 1), for each successive run. Only Run 1 was effective in reducing the inoculated population of *P. fluorescens* Migula below the detection limit (1.0 × 10^1^ cfu/mL; P < 0.05). The lower LTST thermal conditions for Runs 2 and 3 resulted in only 1 log10 reduction. However, in each run for Trial 2, the conditions of the MST unit that followed pasteurization allowed for the significant reduction of all measurable, surviving model microorganisms (P < 0.05). Based on these data, the LTST treatment demonstrated significant reduction of microorganisms present in inoculated milk using optimal operating conditions. Both *L. fermentum* and *P. fluorescens* Migula added to the raw milk at high concentrations (10^6^–10^8^ cfu/mL) were below detection limits after LTST treatment.

### Shelf-life evaluation of LTST treated fluid milk

To determine the shelf stability of LTST treated milk, samples at differing process temperatures were collected after the pasteurizer and the MST unit. These samples were subsequently plated and counted for up to 63 days to determine the shelf-life at 4 °C. Trial 3 microbial count data is shown in Table 2. The process temperatures are also shown in Table 2. The table shows the microbial counts in samples that were run through the pasteurizer (using FDA-required operating conditions) followed by processing through the MST unit. Trial 3 was treated more vigorously with regard to temperature, in that process temperatures were greater than previous trials. The results indicated that, on average, samples taken after the pasteurizer had significant microbial growth after 50 days of storage at 4 °C while all three samples taken after the MST unit had no significant growth after 50 days. Two of the three samples taken after the MST unit continued to have no significant growth after 63 days, at the end of the testing period. The third sample showed growth, but only after 57 days. However, this third sample was treated with only a 1 °C temperature increase within the MST chamber, while samples from runs 1 and 2 were treated with 10 and 5 °C increases, respectively, further demonstrating the effectiveness of the LTST method to prolong shelf-life beyond that of pasteurization.

**Table 2:** Recoverable microorganisms during refrigerated storage at 4 °C

**(A)**								
Microbial growth in samples over time								
Duration (days)	0	21	28	36	43	50	57	63
After pasteurization (cfu/mL)								
Run 1	57^a^	68^a^	9^a^	11^a^	15^a^	5 × 10^2 b^	5 × 10^3 c^	5 × 10^7 d^
Run 2	17^a^	9^a^	<1^a^	3^a^	14^a^	22^a^	3 × 10^5 b^	3 × 10^6 c^
Run 3	<1^a^	54^a^	21^a^	3^a^	8^a^	3 × 10^3 b^	3 × 10^6 c^	3 × 10^7 d^
After MST unit (cfu/mL)								
Run 1	2^a^	3^a^	8^a^	5^a^	7^a^	7^a^	3^a^	<1^a^
Run 2	<1^a^	8^a^	4^a^	5^a^	7^a^	4^a^	4^a^	<1^a^
Run 3	7^a^	6^a^	6^a^	7^a^	5^a^	57^a^	3.02 × 10^2 b^	5 × 10^7 c^

**(B)**						
Microbial growth in samples over time						
Duration (days)	0	7	14	21	28	35
After pasteurization (cfu/mL)						
Run 1	<1^a^	<1^a^	<1^a^	1.67 × 10^4 b^	6.70 × 10^5 c^	1.59 × 10^6 d^
Run 2	1.7^a^	<1^a^	<1^a^	4.33 × 10^3 b^	5.30 × 10^4 c^	7.10 × 10^5 d^
After MST unit (cfu/mL)						
Run 1	<1^a^	<1^a^	<1^a^	7.40 × 10^3 b^	3.57 × 10^5 c^	2.24 × 10^5 c^
Run 2	<1^a^	<1^a^	<1^a^	7.67 × 10^3 b^	1.63 × 10^5 c^	3.80 × 10^5 c^

(C)	Trial 3^1^			Trial 4^1^	
Run					
	1	2	3	1	2
MST Temp In^2^ (°C)	73.0	73.0	73.1	57.2	54.3
MST Chamber (°C)	83.8	78.5	74.4	67.6	64.8
MST Temp Out (°C)	77.5	75.2	70.9	69.0	66.3

^a,b,c, d^Means among the groups were compared using ANOVA and the Tukey’s Test. Within a row, means with a different subscript were different (P < 0.05)

^1^Process temperature (°C) means are of three readings (beginning, middle, and end) of each 30 min total run

^2^MST Temp In is approximately the same as pasteurizer temperature out

Trial 4 was conducted with lower MST processing temperatures to possibly determine a threshold for suitable effectiveness (Table 2B). The data in the table demonstrate that at lower processing temperatures, shelf-life was less stable than that of Trial 3.

The post-pasteurization addition of the LTST method was able to prolong the shelf-life of the product beyond 14 days, referenced to that of conventional pasteurization, to as much as 57 days (Marsili 2000). These data indicated that the LTST method was effective at prolonging shelf-life, utilizing the temperatures listed for Trial 3. Notably, although traditionally pasteurized, the MST process temperatures after were greater in Trial 3, and were still below that of typical pasteurization temperatures and contact times in comparison (Fromm and Boor 2004). An added benefit to this process is that the residual energy from the traditional pasteurizer is utilized for the LTST process, allowing for greater shelf-life without additional energy inputs (Fig. 1).

Overall, shelf-life examination of the pasteurized + MST processed milk samples held at 4 °C showed that no significant microbial growth occurred in samples for up to 57 days when treated at typical, low-end pasteurization temperatures (Fromm and Boor 2004). Additionally, two out of three samples taken after the MST unit continued to have no growth at the end of the testing (63 days). Importantly, Trial 4 demonstrated that at lower temperatures, MST treatment was able to maintain the same effectiveness as pasteurization. Even though shelf-life was not prolonged, lower temperatures from MST treatment were at least as effective as pasteurization. This could prove to be of great benefit when examining process energy inputs.

### Sensory evaluation of LTST pasteurized fluid milk

After determining the effectiveness of the LTST method at reducing microbial load while prolonging shelf-life, it was necessary to determine whether the novel processing technology had an effect on the sensorial characteristics of the milk product. Fifty, untrained panelists were given a chance to comment on likes and dislikes of milk products. Sensory panelists examined color, aroma, taste, aftertaste, and ranking (preference between samples). Table 3A summarizes the sensory comparisons between pasteurized milk and pasteurized + MST treated milk processed during Trial 3. Differences in color, aroma, taste, and aftertaste, were detectable between the samples by panelists as designated in italic (P < 0.05). On three separate occasions, panelists showed no preference between samples produced by either treatment. Sensory evaluations required the raw milk to be pasteurized during Trial 3 to meet FDA regulations, in addition to the MST processing. For this reason, pasteurized samples were compared to pasteurized + MST processed samples. Even under these conditions, sensory panelists either favored pasteurized + MST processed samples or were unable to detect a significant difference in taste and aftertaste compared to traditionally pasteurized milk, with the exception of Run 1. These results were attributed to the process conditions of the run, which had the greatest MST operating temperatures (Table 2C).

**Table 3:** Sensory evaluation of pasteurized and pasteurized + MST processed milk

(A) Trial 3		Sensory characteristics^1^								Preference^2^
Days after processing	Run	Color		Aroma		Taste		Aftertaste		
		P	MST	P	MST	P	MST	P	MST	
21	1	7.49	7.42	*6.36*	*5.83*	*5.62*	*4.60*	*5.38*	*4.21*	*P*
	2	7.16	7.28	5.12	5.30	*4.70*	*4.02*	*4.08*	*3.54*	–
	3	7.28	7.14	5.44	5.40	5.04	5.14	*4.54*	*5.06*	–
28	1	*7.10*	*6.14*	*5.41*	*4.98*	*5.37*	*4.69*	*4.98*	*4.45*	*P*
	2	7.24	7.14	5.38	5.72	*5.02*	*5.72*	4.64	4.96	*MST*
	3	6.90	7.02	5.32	5.40	4.90	5.34	4.74	5.18	*MST*
36	1	*7.14*	*6.40*	5.48	5.28	*5.28*	*4.44*	*4.74*	*3.94*	*P*
	2	7.12	6.86	5.32	5.48	5.18	5.58	4.76	5.02	–
	3	6.73	6.86	5.26	5.24	5.38	5.54	4.82	4.92	*MST*

(B) Trial 5		Sensory characteristics^1^								Preference^3^
	Run	Color		Aroma		Taste		Aftertaste		
		P	MST	P	MST	P	MST	P	MST	
	1&2	7.23	7.26	6.08	6.08	6.86	7.05	6.15	6.38	*MST*

(C)	Trial 5 processing temperatures	
	Run 1^4^	Run 2^4^
MST Temp In^5^ (°C)	N/A	61.7
MST Chamber (°C)	N/A	72.4
MST Temp Out (°C)	N/A	74.2
Pasteurization temp	73.8	73.8

^1^Italic values were different (P < 0.05). Greater values indicate greater preference

^2^Preference; Indicates whether panelists preferred pasteurized sample (P) or pasteurized + MST sample

^3^Preference; Indicates whether panelists preferred pasteurized sample (P) or MST + pasteurized sample

^4^Process temperature (°C) means are of three readings (beginning, middle, and end) of each 30 min total run

^5^MST Temp in is approximately the same as pasteurizer temperature out

Trial 5 was completed using slightly increased processing temperatures (Table 3C) compared to Trial 4 (Table 2C), but was performed using a different process configuration, allowing sensory evaluation of the product with MST + pasteurization. One hundred panelists were used for sensory evaluation. The results in Table 3B show that there were no significant differences in color, aroma, taste, or aftertaste among the untrained panelists. In addition, preference of each treatment was significantly greater for that of MST + pasteurized fluid milk. These results represent similar trends seen with the sensory data from previous trials, and demonstrate that reduction in microbial load using the MST unit, greater than that of traditional pasteurization, has no effect on the sensorial quality of the milk seen with traditional pasteurization.

### Isolation and identification of survivor microorganisms

The effectiveness of the LTST method is dependent upon the physical and thermal mechanisms involved in the process (Arofikin 2010), as well as the microorganisms interrogated. The former has been the focus of the previous pasteurization research, but the latter must also take precedence, which can be accomplished by examining the microorganisms that survive each step in the treatment.

Light scattering technology can be used to differentiate colonies on plated agar that normally would not be differentiated by simple, common, visual identification (Banada et al. 2009). A label-free method called BActerial Rapid Detection using Optical scattering Technology (BARDOT) can be used to aid in the identification of bacterial species by their respective forward scattering patterns (Bae et al. 2011). In this method, the biophysical characteristics of cultured bacteria result in differences of light scattering from a transmitted laser beam, subsequently producing unique scatter images (Bae et al. 2011). Using this automated system, researchers are able to cost-effectively identify unique scatter images, representing the identification and differentiation at the serovar level (Rajwa et al. 2010).

**Fig. 2:**
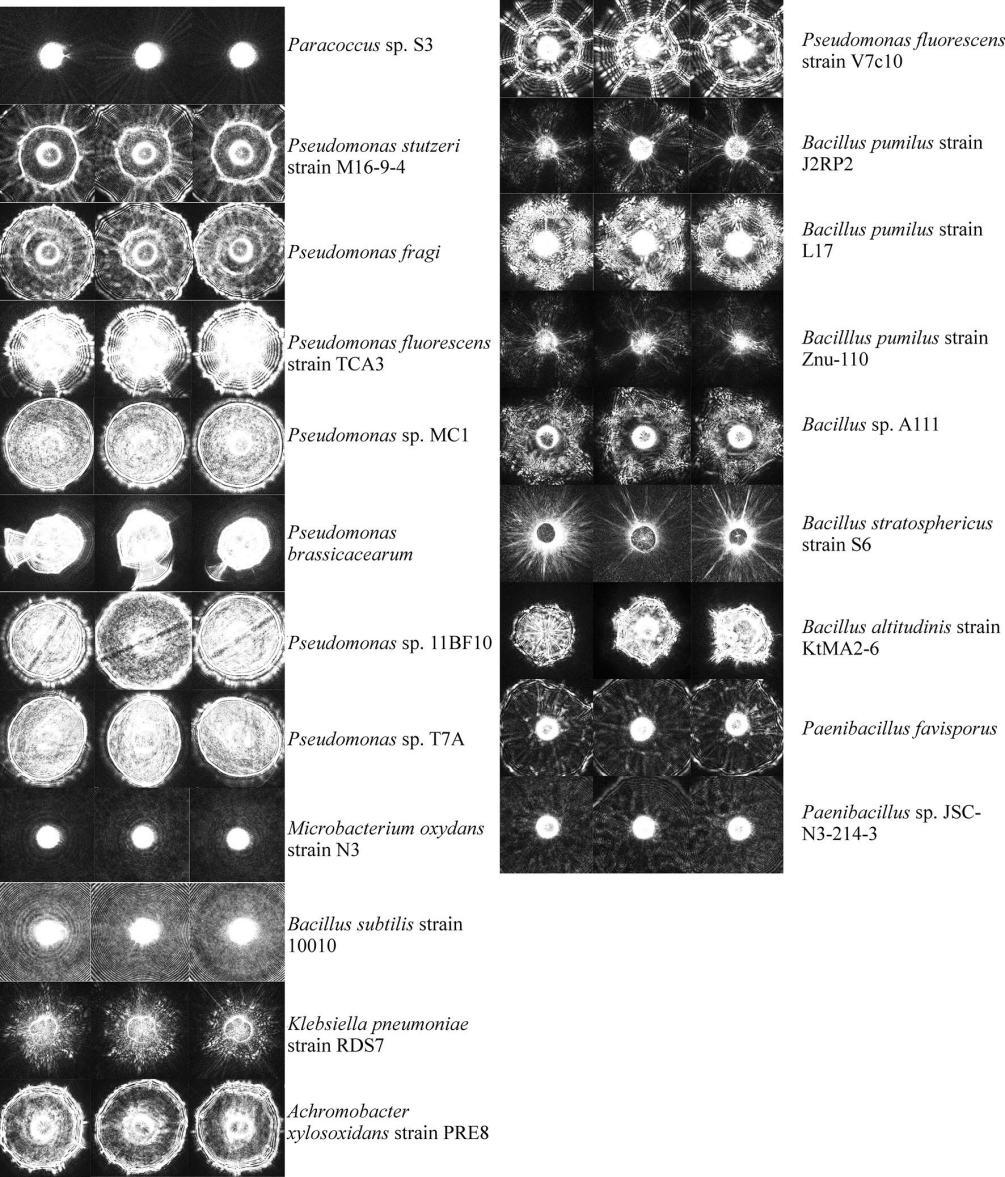
Identification of pasteurization + LTST survivors. Representative scatter images of bacterial species differentiated by BARDOT and identified by 16S rRNA gene sequencing. Survivors were samples from before treatment, after pasteurization, and after LTST treatment

**Fig. 3:**
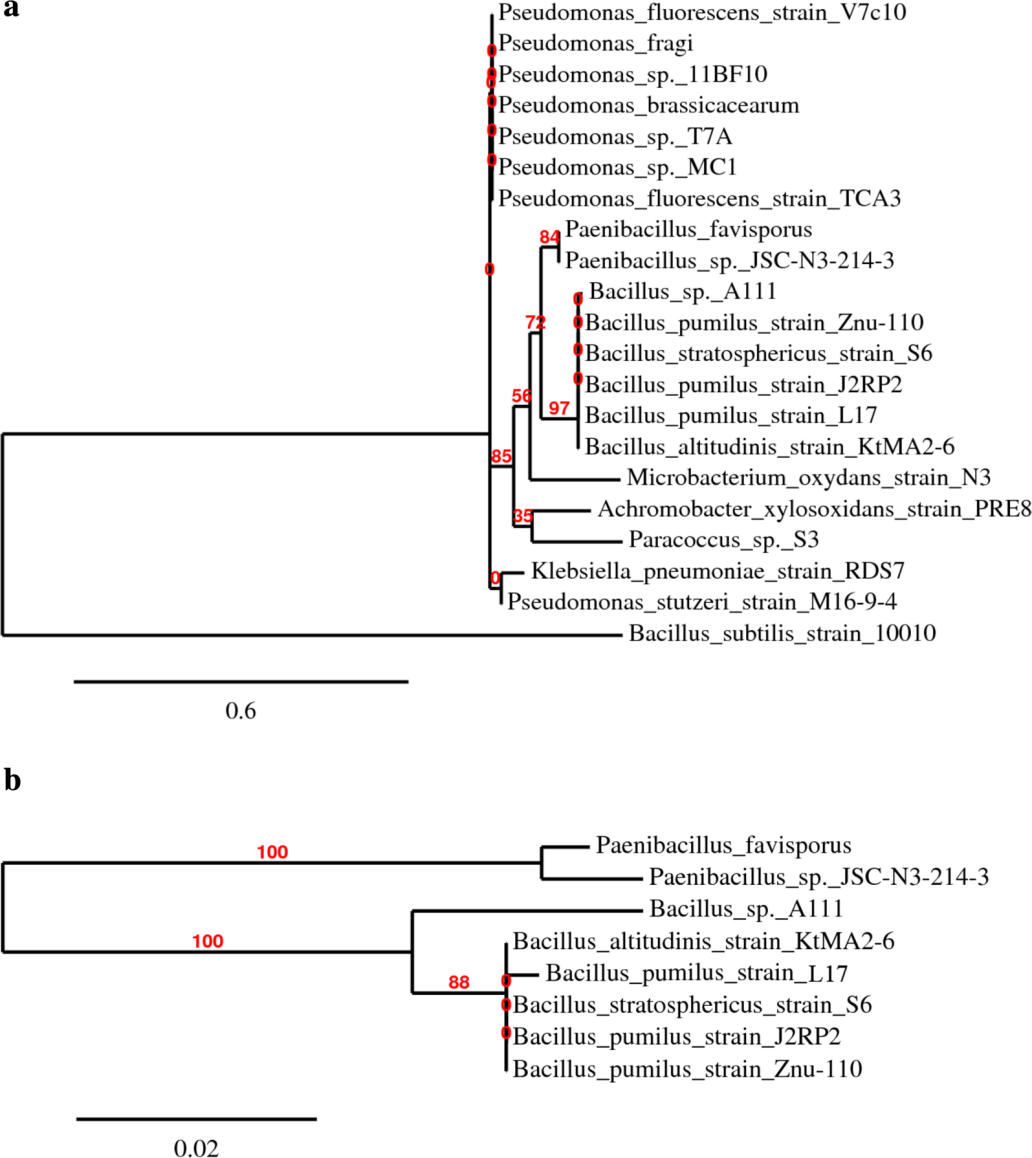
Phylogenetic analysis of microorganisms. Phylogenetic trees displaying the relationships among species identified in the **a** traditionally pasteurized treatment and **b** species surviving the pasteurization + MST treatment. Tree data was determined by the analysis of 16S rRNA gene sequences. The *scale bar* represents substitutions per site. Bootstrap values are shown at the nodes (based on 500 re-samplings). Survivors were sampled from before treatment, after pasteurization, and after pasteurization + MST treatment

Using BARDOT, differing colonies from plated samples within Trial 4 were selected based on their unique scatter images. Each colony, representing potentially different bacterial species, was then identified using 16S rRNA gene sequencing. The bacterial load of the raw milk was of low concentration (~10^2^ cfu/mL) due to its freshness and expedient delivery from the Purdue University Dairy Research and Education Center. The identities and scatter images from BARDOT of the 21 isolated colonies are listed in Fig. 2. All microorganisms were present at the start of processing, but those surviving pasteurization and MST treatment were noted (Table 4) and also ordered as such in Fig. 2. A phylogenetic analysis of organisms present in the pasteurized sample and those surviving pasteurization + MST treatment are depicted in Fig. 3a, b, respectively. Interestingly, many Pseudomonads were identified in the raw sample and after pasteurization, which is typical for dairy products (Cousin et al. 2001). Additionally, an *Achromobacter* species was identified, which is also found in dairy (Poffé and Mertens 1988). However, as predicted, many species that survived traditional pasteurization were from the *Bacillus* or *Paenibacillus* genus, supporting the selection of *L. fermentum* as a model Gram-positive organism for the LTST pasteurization process. Yet, the identification of these spore-forming organisms suggests that assessment of the LTST method needs to be more stringent, by possibly examining the process treatment of heat/stress tolerant *Bacillus* species. Use of such organisms would better serve the evaluation of the LTST method and ultimately allow for easier approval of use over traditional pasteurization methods.

**Table 4:** LTST method and pasteurization survivors designated by sampling location

Organism	Sample location within treatment
*Paracoccus* spp*. S3*	Post-pasteurization
*Pseudomonas stutzeri* strain M16-9-4	Post-pasteurization
*Pseudomonas fragi*	Post-pasteurization
*Pseudomonas fluorescens* strain TCA3	Post-pasteurization
*Pseudomonas* spp*. MC1*	Post-pasteurization
*Pseudomonas brassicacearum*	Post-pasteurization
*Pseudomonas* spp*. 11BF10*	Post-pasteurization
*Pseudomonas* spp*. T7A*	Post-pasteurization
*Microbacterium oxydans* strain N3	Post-pasteurization
*Bacillus subtilis* strain 10010	Post-pasteurization
*Klebsiella pneumoniae* strain RDS7	Post-pasteurization
*Achromobacter xylosoxidans* strain PRE8	Post-pasteurization
*Pseudomonas fluorescens* strain V7c10	Post-pasteurization
*Bacillus pumilus* strain J2RP2	Post-pasteurization + MST
*Bacillus pumilus* strain L17	Post-pasteurization + MST
*Bacilllus pumilus* strain Znu-110	Post-pasteurization + MST
*Bacillus* spp*. A111*	Post-pasteurization + MST
*Bacillus stratosphericus* strain S6	Post-pasteurization + MST
*Bacillus altitudinis* strain KtMA2-6	Post-pasteurization + MST
*Paenibacillus favisporus*	Post-pasteurization + MST
*Paenibacillus* spp*. JSC*-*N3*-*214*-*3*	Post-pasteurization + MST

## Conclusions

The LTST addition demonstrated reduction in microbial load, prolonged shelf-life, and minimal-to-no loss of sensorial/organoleptic properties tested. Still, several parameters of the process need to be examined. Using more thermally robust model microorganisms (e.g. *Bacillus* spp.) will help to better assess process efficacy. Systematic means to better limit product contamination, beyond technological parameters, such as the aforementioned test organisms, would facilitate the application of LTST amendment beyond the laboratory to potential industry applications.

The use of LTST as a method of pasteurization is additionally promising with regard to its source of energy. Currently, FDA regulations require that raw milk be pasteurized. To that end, the MST unit can be connected in-line with a standard pasteurizer to enhance product shelf-life via greater log reduction of spoilage organisms. Current traditional methods of pasteurization have been effective at reducing microbial load by as much as 5.0 log10 cfu/mL (Guan et al. 2005). However, LTST has been able to achieve 7.0–8.0 log10 reduction of microorganisms. Additionally, its in-line connection to a traditional pasteurization tube provides enough energy to run the MST unit, without addition of exogenous heat energy. Thus, the energy-saving characteristics and the previous results serve to demonstrate the effectiveness of the LTST process.

## Methods

### Organisms and growth conditions

*Pseudomonas fluorescens* strain Migula (ATCC Number: 27663) was grown in two, 1L flasks of Luria broth (LB) (pH 7.0–10 % tryptone, 5 % yeast extract, 10 % NaCl), overnight (24 h), with constant aeration at 100RPM, and incubated at room temperature (20–22 °C). The overnight cultures were then used to inoculate ten 16 L carboys containing LB broth (pH 7.0). The inoculated carboys were mixed with magnetic stir bars at room temperature, and allowed to grow for 3 days to ensure a high cellular concentration. Growth was monitored via optical density (OD_600_) measurement with an Eppendorf Biophotometer. Cells were centrifuged at 9803×*g* for 8 min at 4 °C (Beckman Coulter Avanti J-25i) and resuspended in phosphate buffered saline (PBS) (pH 7.0) in order to concentrate the cells from 160 to 2 L and obtain a cellular concentration of 4 × 10^9^ cfu/mL. The concentrated cells were then stored at 4 °C until use the following day. The *P. fluorescens* strain Migula species identity was confirmed by phage typing using Phi-S1.

*Lactobacillus fermentum* was grown in two 1L flasks of Lactobacilli MRS broth (BD Diagnostics, Franklin Lakes, New Jersey) (pH 6.5) overnight, with constant shaking at 100RPM, and incubated at 37 °C. The overnight cultures were then used to inoculate ten 16L carboys containing MRS broth (pH 6.5). The inoculated carboys were mixed using magnetic stir bars at 37 °C, and allowed to grow for 3 days to ensure a high cellular concentration. Cells were centrifuged at 9803×*g* for 8 min at 4 °C and resuspended in phosphate buffered saline (PBS) (pH 7.0) in order to concentrate the cells from 160 to 2 L and obtain a cellular concentration of 1.2 × 10^10^ cfu/mL. The concentrated cells were then stored at 4 °C until use the following day.

### Decimal reduction and thermal death time determination

Decimal reduction and thermal death time were conducted in a water bath, heated by an Isotemp Immersion Circulator (model 730, ThermoFisher Scientific, Waltham, MA), over a magnetic stir plate (Lab-Line Multi-Magnestir, No. 1278, ThemoFisher Scientific, Waltham, MA). Temperature was monitored with American Society for Testing and Materials (ASTM) thermometers accurate to ±0.2 °C. *P. fluorescens* strain Migula and *L. fermentum* were grown overnight and incubated in LB broth (26 °C), and MRS broth (37 °C), respectively, with constant shaking at 100RPM. Experiments were conducted in triplicate. Three 250 mL dual-neck round bottom flasks were filled with 100 mL Grade A ultra-high-temperature (UHT) whole milk (Parmalat, Grand Rapids, MI) and allowed to acclimate to the respective temperature for 10 min; 54.4, 57.2, and 60 °C for *P. fluorescens* strain Migula and 60, 62.8, and 65.5 °C for *L. fermentum*. Flasks were then inoculated with 1 mL of appropriate culture and treated thermally for 5, 10, 20, 40, 80, and 160 s. At the designated time, 1 mL of the sample was extracted from the flask and placed in 9 mL of ice-water-chilled PBS. Samples were allowed to recover for 10 min in an ice water bath. Thermally treated samples were serially diluted in PBS, plated on their respective media agar (LB, MRS), and incubated at their appropriate temperature (26 °C, 37 °C) overnight. Colonies were counted 1 day later and averaged to obtain log reduction values.

The log numbers of the survivors at each time were used to determine D-values. The D-value was determined from the negative reciprocal of the slopes of the regression lines (log10 cfu/mL vs. time of exposure to the thermal treatment, at constant temperature; Mazzola et al. 2003). A linear regression was used from log D-values versus temperature, and the Z-value was obtained from the absolute value of the inverse of the slope.

#### MST processing

The specifics of the MST technology are described in detail in US patent 7,708,941 (Arofikin, 2010). Briefly, the system consists of a balance tank, product pumps, magnetic flow meters, temperature transmitters, level transmitters, tubular heat exchanger with raw regenerator, heater, hold tube, MST chamber, pasteurized regenerator, and cooler sections. The system is started up on water, then sanitized by circulating water at 98 °C for 20 min. After sanitization, the system is cooled down and controlled at the desired operating temperatures for the experiment. The system is equipped with: regenerator bypass on the raw regenerator to allow for control of the temperature out of the regenerator and cascade hot water to heater product out temperature control to control the feed temperature to the MST inlet. The product is then fed to the inlet nozzle or nozzles of the MST chamber at a controlled pressure (pressure = 800,000 Pa). Milk droplets are sprayed into the unit, at which time the milk is then heated to temperature within 0.02 s, which establishes the desired effect. The internal temperature of the MST unit is controlled by the cascade temperature control of hot water to internal temperature. This temperature is controlled at a value 10 °C higher than the inlet temperature. The lower jacket is cooled to prevent further heat treatment of the droplets collecting on the bottom of the MST Chamber. The discharge pump from the MST chamber controls a minimum level in the discharge leg of the MST chamber, and acts as the motive force to push through the downstream pasteurized regenerator and cooling sections of the process, and finally discharged to the destination. The system is controlled for circulation of water, water to product change-over, product discharge, product to water change-over, and clean-in-place (CIP) cycles. The milk is then cooled to 4 °C and expelled from the processor.

### MST-pasteurization milk bacterial reduction

Each trial consisted of three separate runs of homogenized milk from the Purdue University Dairy Research and Education Center (W. Lafayette, IN). Three hundred gallons (~1136L) per run were processed using the tubular pasteurizer followed by the MST chamber (Arofikin 2010). Milk was pumped into a mixing vat and inoculated with 1L of the stored, concentrated *P. fluorescens* Migula or *L. fermentum* (4 × 10^9^ and 1.2 × 10^10^ cfu/mL, respectively). Raw milk bacterial load was 5.7 × 10^4^ (±1.5 × 10^3^) cfu/mL. The inoculated milk was then pumped into the holding basin within the pasteurization unit and processed. The duration of each run was approximately 30 min. Samples of milk were collected at 3 time points (10 min intervals) throughout the run, and at locations before processing, after the pasteurizer, and after the MST chamber. Before extraction, sterile sample diaphragms were attached to collecting ports, and were additionally treated with 70 % ethanol. Milk was extracted and collected via sterile 250 mL QMI sampling assembly bags (QMI, Oakdale, MN), with an 18 gauge needle and three feet of tubing. After the sampling bag was filled, the needles were removed from the port and capped with a Luer lock fitting and cap. Collected milk was immediately stored in an ice water bath and collecting ports were again sterilized with 70 % ethanol.

Samples from the processed runs were aseptically drawn, diluted in phosphate buffered saline (PBS, pH 7), plated, and incubated on LB agar (17 %, 26 °C) and Lactobacilli MRS agar (17 %, 37 °C) to determine the bacterial reduction of *P. fluorescens* Migula and *L.fermentum,* respectively. Samples were stored at 4 °C between plating periods. Samples were plated weekly for 5 weeks (35 days) to track the growth of the inoculated runs. Plates were counted after 2 days of growth.

### Shelf-life determination

Experimental runs were completed without bacterial inoculation in order to assess the shelf-life of the milk product and to perform sensory evaluation. Processing was conducted similarly to the previous method; albeit without the bacterial mixing vat. Extraction and collection of the processed milk were performed similarly to the previous procedures (conditions are previously noted). In addition, milk was collected after pasteurization alone and pasteurization + MST and into sterile, 2 L, brown glass bottles for sensory evaluation. The bottles were housed in a laminar flow hood in order to prevent post-processing contamination. After extraction, the bottles were stored at 7 °C until sensory evaluation.

Samples from the raw milk processed runs were aseptically drawn, diluted in phosphate buffered saline (PBS, pH 7), plated on plate count agar (PCA, HiMedia Laboratories, Mumbai, India), and incubated at both 26 and 7 °C to assess psychrophile survivability and growth. Samples were stored at 4 °C between plating periods. Samples were plated weekly for 5–9 weeks (35–63 days), dependent upon the study, to determine the shelf-life of the processed raw milk. Plates incubated at 26 °C were counted after 2 days of growth, while plates incubated at 7 °C were counted after 5 days of growth.

### Sensory evaluation

Panelists participated voluntarily. This study was approved by the local research ethics committee (IRB Protocol Number 1209012647). Sensory evaluation using a paired comparison test of 50 untrained panelists was performed. Samples from pasteurized, pasteurized + MST, and MST + pasteurized treated milk were compared for preference/acceptability. Testing was performed 21, 28, and 36 days after processing. The products were kept in refrigeration when not in use (7 °C). Prior to testing, the milk was poured into a pitcher and a hand blender was used to further homogenize the milk. Each panelist was given a 2 sample in a 5 oz. drinking cup and was first asked hedonic and ranking questions. The panelists were given a chance to comment on likes and dislikes. Parameters examined were color, aroma, taste, aftertaste, and ranking.

Multiple comparison tests were conducted. Tukey’s HSD was performed to control for maximum experiment-wise error rate and can be used without F protection. According to standard practice, LSD and Duncan’s were only considered if the ANOVA P value was deemed acceptable to control for experiment-wise error rates (under the complete null hypothesis). If Duncan’s Multiple Range Test was used, only the largest critical range was reported. If automatic significance was selected, an available significance level was chosen for the multiple comparison test based on the observed P value.

### Application of BARDOT to detect and identify bacterial species

Growth on plates from representatives of the raw processed runs were initially differentiated by BARDOT (Banada et al. 2009), selected, serially diluted in PBS and plated on PCA to obtain isolated colonies, then incubated at 26 °C for 24 h or until colony size reached 1.3 ± 0.2 mm. Colony size was used as a fixed parameter because growth rates are variable among species. Scatter images of colonies were acquired and analyzed using the BARDOT system (Advanced Bioimaging Systems, W. Lafayette, IN). Unique scatter images were selected for identification by 16S rRNA gene sequencing.

### Bacterial identification by 16S rRNA gene sequencing

Cultures were identified by 16S rRNA gene sequencing of PCR-amplified products (Lane 1991; Marchesi et al. 1998). 16S rRNA-specific primer pairs, 27F and 1492R were used to amplify the target gene 1465 bp in length. PCR conditions include: an initial denaturation at 94 °C for 4 min, followed by 30 cycles consisting of 94 °C for 55 s, 46 °C for 55 s, and 72 °C for 4 min, and final extension at 72 °C for 9 min. PCR products were sequenced, quality checked, and cleaned by the Purdue Genomics Core Facility (Purdue University, W. Lafayette, IN), and the 16S reads were classified using the NCBI nucleotide collection database. Phylogenetic analysis was conducted from the resultant sequences and analyzed via alignment, curation, phylogeny, and tree rendering programs from Dereeper et al. (2008).

## Additional file

10.1186/s40064-016-2250-1 D- and Z-values for *P. fluorescens* Migula. **Figure S2.** D- and Z-values for *L. fermentum*. **Figure S3.** Sampling model.

## Abbreviations

BARDOT: BActerial Rapid Detection using Optical scattering Technology
HPP: high-pressure processing
LTLT: low temperature-long-time
LTST: low temperature-short time
MST: Millisecond Technologies
UHT: ultra-high-temperature
