# Supplementary material for: The effect of a novel low temperature-short time (LTST) process to extend the shelf-life of fluid milk
Source: Springerplus. 2016 May 31;5(1):660. doi: 10.1186/s40064-016-2250-1 (PMC4899401; doi:10.1186/s40064-016-2250-1)

**Supplemental Material**

**The Effect of a Novel Low Temperature-Short Time (LTST) Process to Extend the Shelf-Life of Fluid Milk**

Phillip R. Myer ^1^, Kyle R. Parker ^2^, Andrew T. Kanach ^2^, Tengliang Zhu ^3^, Mark T. Morgan ^4^, and Bruce M. Applegate ^2, 3*^

Running Title: Low Temperature-Short Time Processing

^1^ Department of Animal Science, University of Tennessee, Knoxville, TN 37996

^2^ Department of Biological Sciences, Purdue University, West Lafayette, IN 47907-2054

^3^ Department of Food Science, Purdue University, West Lafayette, IN 47907–2009

^4^ Food Science and Technology Department, University of Tennessee, Knoxville, TN 37996

Email: PRM, pmyer@utk.edu; KRP, kyparker@gmail.com; ATK, akanach@purdue.edu, TZ ztl20082002@hotmail.com; MTM, Mark.Morgan@utk.edu; BMA, applegate@purdue.edu

***** Corresponding Author: applegate@purdue.edu, (765) 496-7920

**Additional file 1: Figure S1** D- and Z-values for *P. fluorescens* Migula. **Figure S2** D- and Z-values for *L. fermentum*. **Figure S3** Sampling Model.

**Figure S1** D- and Z-values for *P. fluorescens* Migula. A) D-values at 54.44, 57.22, and 60°C. B) Z-value. C) Table summarizing the above information for gram reaction, decimal reduction time, and z-value.

A)

B)

C)

**Figure S2** D- and Z-values for *L. fermentum*. A) D-values at 60, 62.78, 65.56°C. B) Z-value. C) Table summarizing the above information for gram reaction, decimal reduction time, and z-value.

A)

B)

C)

**Figure S3** Sampling Model. Sampling occurred at three locations within the unit and at 3 defined sampling times within each run. Locations included the holding tank (raw milk, or inoculated milk), after pasteurization, and after MST+pasteurization. Method depiction is general and may differ where noted.


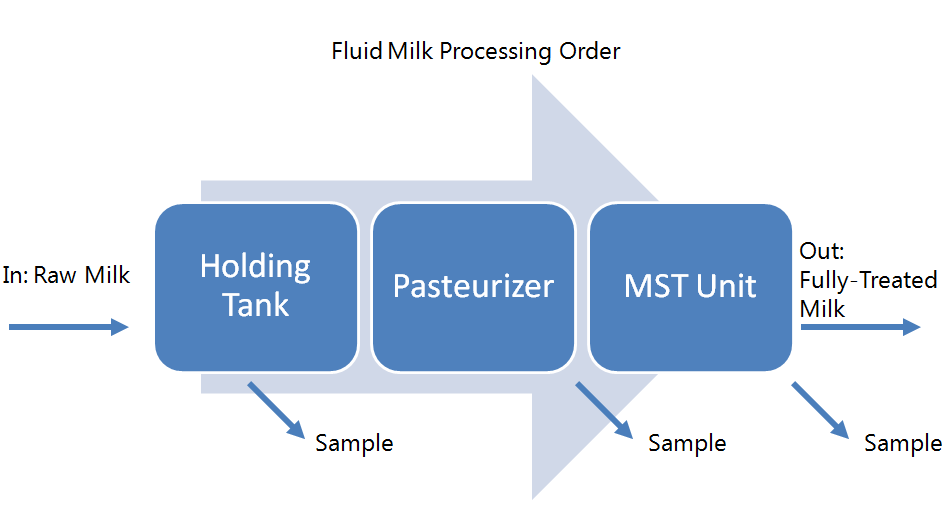

Supplement: Supplementary file 1 — 10.1186/s40064-016-2250-1 D- and Z-values for P. fluorescens Migula. Figure S2. D- and Z-values for L. fermentum. Figure S3. Sampling model. [file 40064_2016_2250_MOESM1_ESM.doc]
